# The Host CYP1A1-Microbiota Metabolic Axis Promotes Gut Barrier Disruption in Methicillin-Resistant *Staphylococcus aureus*-Induced Abdominal Sepsis

**DOI:** 10.3389/fmicb.2022.802409

**Published:** 2022-04-27

**Authors:** Xiaoyuan Ma, Huaijian Jin, Xiang Chu, Weihong Dai, Wanqi Tang, Junyu Zhu, Fangjie Wang, Xue Yang, Wei Li, Guodong Liu, Xia Yang, Huaping Liang

**Affiliations:** ^1^State Key Laboratory of Trauma, Burns and Combined Injury, Department of Wound Infection and Drug, Daping Hospital, Army Medical University, Chongqing, China; ^2^Department of Spine Surgery, Center of Orthopedics, Daping Hospital, Army Medical University, Chongqing, China; ^3^Trauma Center, The Second Affiliated Hospital of Hainan Medical University, Haikou, China; ^4^State Key Laboratory of Trauma, Burn and Combined Injuries, Medical Center of Trauma and War Injuries, Daping Hospital, Army Medical University, Chongqing, China

**Keywords:** CYP1A1, gut microbiota, intestinal barrier, cadaverine, enterocytes junction, sepsis, methicillin-resistant *Staphylococcus aureus*

## Abstract

**Background:**

Host-microbiota crosstalk has been implicated in multiple host metabolic pathway axes that regulate intestinal barrier function. Although constitutive cytochrome P4501A1 (CYP1A1) expression perturbs the microbiome-derived autoregulatory loop following enteric infection, little is known about the role of host CYP1A1 in modulating gut microbiome-mediated signaling during methicillin-resistant *Staphylococcus aureus* (MRSA)-induced abdominal sepsis and its effects on intestinal barrier integrity.

**Methods:**

Abdominal sepsis was induced by the intraperitoneal injection of MRSA in mice. The effect of CYP1A1 deficiency on gut barrier integrity was investigated using RNA sequencing, microbiome analyses, and targeted metabolomics. The microbiota-produced metabolites were validated in patients with sepsis and persistent MRSA infection.

**Results:**

Mice lacking CYP1A1 exhibited an altered gut microbiome, a reduced metabolic shift from lysine to cadaverine in the caecal contents and antimicrobial molecule production (Retnlb, Gbp7, and Gbp3), and they were protected against gut barrier disruption when subjected to MRSA challenge. These beneficial effects were validated in aryl hydrocarbon receptor (AHR) knockout (KO) mice by cohousing with CYP1A1 KO mice and abrogated after supplementation with cadaverine or *Enterococcus faecalis*, the primary microbiota genus for cadaverine synthesis. Antibiotic-driven gut dysbacteriosis impaired the survival benefit and disrupted the intestinal barrier integrity in CYP1A1 KO mice after MRSA infection. Furthermore, increased cadaverine levels in feces and serum were detected in critically ill patients with gut leakiness during persistent MRSA infection, whereas cadaverine was not detected in healthy controls. Additionally, microbiota-derived cadaverine induced enterocyte junction disruption by activating the histamine H4 receptor/nuclear factor-κB/myosin light-chain kinase signaling pathway.

**Conclusion:**

This study revealed the unexpected function of host CYP1A1 in microbiota-mediated cadaverine metabolism, with crucial consequences for dysbacteriosis following MRSA-induced abdominal sepsis, indicating that inhibiting CYP1A1 or blocking cadaverine-histamine H4 receptor signaling could be a potential therapeutic target against abdominal sepsis.

**Clinical Trial Registration:**

[http://www.chictr.org.cn/index.aspx], identifier [ChiCTR1800018646].

## Introduction

Sepsis is a multifactorial syndrome caused by systemic or localized infections, leading to life-threatening organ dysfunction (Cheung et al., 2021). Abdominal sepsis is attributed to primary or secondary peritonitis triggered by Gram-positive or Gram-negative bacteria (Martin-Loeches et al., 2019), including methicillin-resistant *Staphylococcus aureus* (MRSA). Abnormal MRSA colonization in the gut markedly increases the risk of MRSA-associated enteritis, and the resistance gene can be transferred to commensal microbes (Huttner et al., 2012; Nataraj and Mallappa, 2021). Paradoxically, combination antibiotic therapy for MRSA salvage treatment will aggravate intestinal disorders. Furthermore, antibiotics do not exert their effect in a vacuum but within a complex host (Kullar et al., 2016). Thus, using novel host-based therapeutic strategies to remodel the health-promoting gut microbiota for MRSA-induced dysbacteriosis is a promising direction.

The gut microbiota composition is subject to the host genome from birth and participates in multiple host metabolic pathway axes that regulate intestinal barrier function (Nicholson et al., 2012; Sharkey et al., 2018). Gut barrier integrity is seriously disrupted during severe peritonitis-induced sepsis (Zhou and Verne, 2018). Thus, the abdominal sepsis-induced dysbiotic intestinal microenvironment leading to increased epithelial permeability and gut barrier disruption may facilitate pathogenic bacterial colonization. Specifically, as a gatekeeper against bacterial translocation, the intestinal epithelial barrier relies on enterocyte junctions to maintain integrity (Sharkey et al., 2018). However, a gap exists in understanding the host–microbiota interaction involved in regulating the intestinal epithelial cell (IEC) junctions during abdominal sepsis. Notably, constitutive expression of cytochrome P4501A1 (CYP1A1) throughout intestinal epithelial cells increases the susceptibility to enteric infection (Schiering et al., 2017), and whether the host CYP1A1–gut microbiota axis affects IEC barrier disruption against abdominal sepsis remains unclear.

Our previous study demonstrated that CYP1A1 mRNA expression levels are elevated in PBMCs of patients with sepsis, and strongly correlate with sequential organ failure assessment (SOFA) scores (Tian et al., 2020). Similarly, polymorphisms in the CYP1A1 gene partially contribute to the increased vulnerability to community-acquired pneumonia (CAP) and nosocomial pneumonia (NP) (Salnikova et al., 2013; Zhao et al., 2017). Compounds that inhibit the activity or expression of CYP1A1 exert anti-inflammatory, antitumor, anti-*mycoplasma*, anti-*Citrobacter rodentium*, and anti-parasitic effects (Westerink et al., 2008; Wincent et al., 2012; Fang et al., 2016). Furthermore, germ-free mice (microbiota deficient from birth) or those treated with antibiotics demonstrated a lower level of CYP1A1 expression (Korecka et al., 2016; Fu et al., 2017; Wang et al., 2018). Diurnal 6-formylindolo[3,2-b]carbazole/aryl hydrocarbon receptor (AHR)/CYP1A1 feedback controls the intestinal immune system to coordinate the diurnal rhythmicity of gut microbiota (Rannug, 2020). In this context, unsurprisingly, a crosstalk between CYP1A1 and the resident gut microbiome may exist. Several studies have suggested that constitutive CYP1A1 expression rapidly metabolizes AHR ligands in the gut lumen, indirectly impairing AHR activation in the intestinal immune system following enteric infection (Schiering et al., 2017; Stockinger et al., 2021). Intriguingly, we previously reported the possible AHR-independent regulation of CYP1A1 expression and CYP1A1-induced proinflammatory responses during the progression of inflammation and sepsis (Tian et al., 2020). However, we did not explore whether the interaction between CYP1A1 and microbe-mediated signaling is independent of AHR. Thus, the possibility of non-AHR-dependent action on the intestinal barrier integrity in MRSA-induced abdominal sepsis remains to be determined.

In the present study, we sought to investigate the role of host CYP1A1 as a gut flora modulator and its impact on microbiota-mediated metabolic signaling during abdominal sepsis-induced intestinal epithelial disruption. Our data showed that compared to *Cyp1a1*^+/+^ mice, *Cyp1a1*^–/–^ mice showed attenuated acute MRSA-induced gut barrier disruption, partially explained by the absence of severe microbial dysbiosis and harmful gut microbial metabolites. We further demonstrated that the increased abundance of *Enterococcus faecalis* impaired MRSA-induced intestinal barrier dysfunction, primarily *via* cadaverine production and the degradation of IEC junctions. Furthermore, host CYP1A1–microbiota crosstalk involving the gut microbiome-cadaverine-enterocyte junction axis could be transferred to AHR KO mice through microbiota horizontal transmission.

## Materials and Methods

### Animal Experiments

Male C57BL/6 mice [wild-type (WT), 6–8 weeks old, 20–23 g] were obtained from the Experimental Animal Center of Army Medical Center. Whole-body *Cyp1a1*-knockout (KO) (*Cyp1a1*^–/–^) mice with a C57BL/6 background were born from heterozygotes at the Army Medical Center of PLA (Chongqing, China) transgenic animal breeding facility. The mice were separated into different cages based on their genotype (*Cyp1a1*^+/+^, *Cyp1a1*^±^, and *Cyp1a1*^–/–^) and sex after weaning. Two or three mice were housed in the same cage following same-sex and same-genotype feeding rules. Considering the existence of parental effects, littermates of the same sex and genotype were randomly assigned to different cages. Male C57BL/6 *Ahr*^±^ mice were purchased from the Jackson Laboratory. The *Ahr*^–/–^ mice were born from heterozygotes at our facility. To assess gut microbiota-mediated signaling in a non-AHR-dependent manner in *Cyp1a1*^–/–^ mice, we performed a cohousing experiment. The *Ahr*^–/–^ mice were cohoused with *Cyp1a1*^+/+^ mice and *Cyp1a1*^–/–^ mice at a 1:1 ratio after weaning for 10 weeks. All the mice were housed in autoclaved cages under a 12 h light/dark cycle and 25°C conditions. The reagents used and their concentrations for the *in vivo* experiments were as follows: cadaverine (250 mg/kg; Sigma–Aldrich, St. Louis, MO, United States) and JNJ7777120 (150 mg/kg; Selleck, Houston, TX, United States). The mice were treated daily with 4 × 10^7^ colony-forming units (CFUs) of *E. faecalis* (ATCC47077, Manassas, VA, United States) by oral gavage. The mouse experiments were approved by the Laboratory Animal Welfare and Ethics Committee of Third Military Medical University (AMUWEC20201455).

### Methicillin-Resistant *Staphylococcus aureus*-Induced Peritonitis in a Mouse Abdominal Sepsis Model

The MRSA strain was obtained from a clinical laboratory of the Army Medical Center of PLA. The MRSA was routinely incubated in 15 ml of Luria-Bertani liquid medium (10 g/L of NaCl, 5 g/L of Yeast Extract, and 10 g/L of tryptone) on a shaker at 37°C overnight at 200 rpm. The bacterial solution was collected the next day, and each well was detected at 570 nm after doubling dilution using an enzyme-labeled instrument (BioTek, Winooski, VT, United States) to determine the concentration. The MRSA [3 × 10^8^ CFUs/mouse] suspended in sterile saline was injected intraperitoneally into mice to establish the abdominal sepsis model.

### Human Subjects

Human blood and fecal sample collection and the study procedures were approved by the Ethics Committee of Army Medical Center of PLA, China (ChiCTR1800018646), and informed consent forms were completed by all the patients and healthy volunteers. For this prospective clinical study, 18 patients were diagnosed with sepsis according to 2016 Surviving Sepsis Campaign guidelines (Rhodes et al., 2017), and 20 healthy controls were recruited after screening at the Army Medical Center of PLA from December 2019 to January 2021. All patients with sepsis met the following criteria: (1) age ≥ 18 years; (2) length of ICU stay ≥ 48 h; (3) patients with continuous indwelling catheters, and MRSA detected in specimens submitted within 48 h. Patients who abandoned the treatment were transferred to another hospital or had a clinical infectious disease (e.g., HBV and HIV infection) were excluded from this study. Healthy control subjects were enrolled in the Physical Examination OPC of Army Medical Center for blood and fecal sample collection. For all patients with sepsis and MRSA infection, a dynamic collection of samples was conducted during the infection period. Whole blood samples were quickly sent to the laboratory for serum extraction within 2 h after collection, and fecal samples were placed in liquid nitrogen immediately.

### Pathogen Load Detection

The method to determine the bacterial load in the peripheral blood has been described previously (Tian et al., 2020). Briefly, blood was drawn from the caudal vein of the mice 12 h after MRSA challenge (*n* = 3/group) and was diluted 100-fold with 1 ml of sterile Luria-Bertani agar plates. The number of bacterial colonies, CFUs, was counted after 18 h of sterile incubation at 37°C.

### Intestinal Epithelial Cell Preparation

Primary intestinal epithelial cells (IECs) were carefully isolated from mice according to the established protocol provided by Dávalos-Salas et al. (2019). Briefly, the ileum opened longitudinally were washed in cold 1 × phosphate-buffered saline (supplemented with 1 mM of phenylmethylsulfonyl fluoride and 1% of penicillin-streptomycin solution) in a 10 cm Petri dish and moved to a 50 ml centrifuge tube with tissue digestion buffer (5% of fetal bovine serum, Hanks’ Balanced Salt Solution, 15 mM of EDTA, and RNA later) by incubation in a 37°C shaker, at 200 rpm, and for 30 min. The tissues were then transferred to a preheated collagenase XI, neutral protease I, and DNase I solution in a 37°C shaker for 20 min. IEC pellets were collected after centrifugation for 5 min at 4°C at 1,200 rpm.

### Ileum Transcriptome Sequencing and Analysis

Total RNA was extracted from the ileal tissues of the *Cyp1a1*^+/+^_con, *Cyp1a1*^+/+^_MRSA, *Cyp1a1*^–/–^_con, and *Cyp1a1*^–/–^_MRSA groups using TRIzol (Invitrogen, Carlsbad, CA, United States) according to the manufacturer’s instructions, and each group was prepared using four parallel replicates. RNA sequencing was performed by the Beijing Genomics Institute Sequencing and Microarray Facility using the BGIseq500 platform (BGI-Shenzhen, China). Differential expression analysis was performed using DESeq2 (v1.4.5) (Love et al., 2014) with a *Q*-value ≤ 0.05. To perform gene set analysis, Gene Ontology (GO)^1^ and Kyoto Encyclopedia of Genes and Genomes (KEGG)^2^ enrichment analyses of annotated differentially expressed genes (DEGs) were performed using Phyper^3^ based on the hypergeometric test. The significance levels of terms and pathways were corrected using the *Q*-value with a rigorous threshold (*Q*-value ≤ 0.05) and the *q*-value calibration package^4^. All the data were analyzed using the online Dr. Tom network platform of BGI^5^.

### Ultra-High-Performance Liquid Chromatography Analysis of the Cadaverine Concentration in the Serum and Caecal Contents

All the samples from humans and mice were extracted and derivatized as reported previously (Liu et al., 2013; Levy et al., 2015) with a few modifications. Briefly, a 100 μL aliquot of the clear supernatant (or standard solution) was transferred to an Eppendorf tube and then mixed with 50 μL of 20 mg/mL of dansyl chloride in acetonitrile and 50 μL of 0.1 mol/L sodium carbonate after a 1 h incubation at 40°C in the dark. Dansyl derivatives were added to 50 μL of 0.1% of formic acid in water, vortexed for 15 s, and centrifuged at 12,000 rpm and 4°C for 10 min. An 80 μL aliquot of the clear supernatant was transferred to an autosampler vial for UHPLC–MS/MS analysis. The UHPLC separation was performed using an Agilent 1290 Infinity II series UHPLC system (Agilent Technologies, Santa Clara, CA, United States) equipped with a Waters ACQUITY UPLC HSS T3 column (100 × 2.1 mm, 1.8 μm). Mobile phase A was 10 mmol/L ammonium formate and 0.1% of formic acid in water, and mobile phase B was acetonitrile. The column temperature was set at 35°C. The autosampler temperature was set at 4°C, and the injection volume was 2 μL. An Agilent 6460 triple quadrupole mass spectrometer (Agilent Technologies, Santa Clara, CA, United States) equipped with an AJS electrospray ionization interface was applied for assay development. Agilent MassHunter Workstation Software (B.08.00; Agilent Technologies, Santa Clara, CA, United States) was used for MRM data acquisition and processing. The signal-to-noise ratios (S/Ns) were used to determine the lower limits of detection and quantitation. The precision of the quantitation was measured as the relative standard deviation, determined by injecting analytical replicates of a quality control (QC) sample. The accuracy of quantitation was evaluated by the analytical recovery of the QC samples. The percent recovery was calculated as [(mean observed concentration)/(spiked concentration)] × 100%.

### Microbial Biosynthesis of Cadaverine (*LdcC* and *CadA*)

Caecal content samples were collected immediately after sacrifice in RNase-free cryotubes, snap-frozen in liquid nitrogen, and stored at −80°C. Total caecal microbial DNA was extracted using the QIAamp^®^ Fast DNA Stool Mini Kit (51604, Inc., Duesseldorf, Germany) according to the manufacturer’s instructions. The abundance of *LdcC* and *CadA* from mouse caecal DNA samples were determined by quantitative polymerase chain reaction (qPCR). The *LdcC* gene was amplified using the forward primer CGGCCCTTATAACCTGCTGTTTC and reverse primer CC TTGTGCCAGATCCTGAATACG. The *CadA* gene was amplified using the forward primer GTCTGTGCGGCGTTATTTTTGAC and reverse primer CACCCAGCGCATATTCAAAGAAG.

### 16S rDNA Sequencing and Analysis

Caecal content samples were collected immediately after sacrifice in RNase-free cryotubes, snap-frozen in liquid nitrogen, and stored at −80°C. Total caecal microbial DNA was extracted using the E.Z.N.A. ^®^Stool DNA Kit (D4015; Omega, Inc., Alpharetta, GA, United States) according to the manufacturer’s instructions. The DNA extraction quality and concentration were detected by 1% agarose gel electrophoresis and UV spectrophotometry. The 16S rDNA genes were amplified using specific primers with barcodes on the 5′ ends targeting the V3–V4 region (Logue et al., 2016): 341 forward primer CCTACGGGNGGCWGCAG – 805 reverse primer GACTACHVGGGTATCTAATCC. The PCR products were purified using AMPure XT beads (Beckman Coulter Genomics, Danvers, MA, United States) and quantified using Qubit (Invitrogen, Carlsbad, CA, United States). The size and quantity of the amplicon library were assessed using an Agilent 2100 Bioanalyzer (Agilent, Santa Clara, CA, United States) and the Library Quantification Kit for Illumina (Kapa Biosystems, Woburn, MA, United States), respectively. The libraries were sequenced using the NovaSeq PE250 platform. The sequencing depth was approximately 50,000 tags. Quality filtering of the raw reads was performed under specific filtering conditions to obtain high-quality clean tags according to fqtrim (v0.94). Chimeric sequences were filtered using Vsearch software (v2.3.4). Paired-end reads were assembled using fast length adjustment of short reads (FLASH, v1.2.8). Alpha diversity and beta diversity were calculated by normalizing to the same sequences randomly using QIIME2. Next, according to the SILVA (release 132) classifier, feature abundance was normalized using the relative abundance of each sample. BLAST (NCBI, United States) was used for sequence alignment, and the feature sequences were annotated using the SILVA database for each representative sequence. The total raw read counts and processed read counts for all samples were 27,72,681 and 22,86,029, respectively.

### Diamine Oxidase and Lysine Decarboxylase Activity Quantification

Serum from humans was used to detect diamine oxidase (DAO) enzyme activity using enzyme-linked immunosorbent assay (ELISA), as described by Honzawa et al. (2011). The human blood samples were kept at room temperature for 1 h before centrifugation for 20 min at 1,000 × *g* and were immediately stored at −80°C for subsequent quantification.

After sonication of the mouse caecal contents, the intestinal bacterial cell supernatant was used to determine lysine decarboxylase (LDC) enzyme activity using ELISA, as previously described (Lee and Cho, 2001). Feces samples (100 mg) suspended in 2.5 ml of cold phosphate-buffered saline were centrifuged at 1,000 × *g* for 20 min to obtain the supernatant and were centrifuged again (4,000 rpm, 20 min, 4°C) to obtain the bacterial cells. These cells resuspended in lysis solution (50 mM of Tris-HCl at pH 7.4, 2 mM of EDTA, and 100 mM of NaCl) were crushed using an ultrasonic cell disruptor (BRANSON, Branson, MO, United States) under the following conditions: 20 kHz, 150 W, and crushing for 60 s three times, 60 s apart at 4°C.

### Fluorescein Isothiocyanate-Dextran Permeability Assay of the Ileum

*In vivo* leakage of mouse intestinal barrier function was measured using fluorescein isothiocyanate (FITC)-dextran (FD4; Sigma–Aldrich, St. Louis, MO, United States) at a 500 mg/kg dose as described previously (Natividad et al., 2018). The mice were orally administered 0.3 ml of FD4, 8 h before sacrifice, and serum samples were dynamically collected 6, 12, and 24 h after MRSA-induced sepsis. The FITC-dextran levels in serum were determined using a fluorescence microplate reader (BioTek, Winooski, VT, United States), setting an excitation wavelength of 470 nm and emission wavelength of 530 nm.

### Immunofluorescence Analysis

The method for cell immunofluorescence was described previously (Zhu et al., 2018). The primary antibodies used were mouse anti-CYP1A1 (Santa Cruz; sc-25304; 1:100) and mouse anti-AHR (Gene Tex; GTX22770; 1:100). The method for ileum tissue immunofluorescence was described previously (Kumar et al., 2021). The primary antibodies used were rabbit anti-ZO-1 (Invitrogen; PA5-85256; 1:100) and rabbit anti-occludin (Invitrogen; 40-4700; 1:100). The secondary antibodies used were goat anti-Mouse IgG (H + L) highly cross-adsorbed secondary antibodies (Invitrogen; Alexa Fluor Plus 488 and 647; 1:400). All the images were captured using an FV3000 confocal microscope (Olympus, Tokyo, Japan).

### Histopathological Examination

Ileum tissues were sectioned at 5 μm of thickness and stained with hematoxylin and eosin (H&E) using standard protocols. The histological scores were evaluated using a scoring system. The sections primarily contained inflammatory cell infiltrate, epithelial damage, and mucosal architecture (Martin et al., 2021).

### Quantitative Reverse Transcription PCR

Total RNA was extracted using the PureLink RNA Kit (Invitrogen, Carlsbad, CA, United States; 12183018A). The cDNA was synthesized by reverse transcription using the PrimeScript RT Reagent Kit (Takara Biotechnology Co. Ltd., Tokyo, Japan). The expressions of CYP1A1, zonula occludens (ZO)-1, occludin, Retnlb, Gbp-3, Gbp-7, HRH1, HRH2, HRH3, HRH4, and glyceraldehyde-3-phosphate dehydrogenase (GAPDH) mRNAs were amplified and quantified in triplicate using 1 μg of cDNA and SYBR Premix (TaKaRa Bio, Tokyo, Japan) and the BioRad CFX96 biosystem (Table 1).

**TABLE 1:** Primers used in the RT-qPCR reactions.

Gene Symbol	Murine forward primer (5′–3′)	Murine reverse primer (5′–3′)
CYP1A1	GACCCTTACAAGTATTTGGTCGT	GGTATCCAGAGCCAGTAACCT
ZO-1	GCCGCTAAGAGCACAGCAA	TCCCCACTCTGAAAATGAGGA
Occludin	TTGAAAGTCCACCTCCTTACAGA	CCGGATAAAAAGAGTACGCTGG
Retnlb	AAGCCTACACTGTGTTTCCTTTT	GCTTCCTTGATCCTTTGATCCAC
Gbp-3	ACATGCAGATTCCACTTCCAAA	CCGTCCTGCAAGACGATTCA
Gbp-7	TCCTGTGTGCCTAGTGGAAAA	CAAGCGGTTCATCAAGTAGGAT
HRH1	CAAGATGTGTGAGGGGAACAG	CTACCGACAGGCTGACAATGT
HRH2	CCCAATGGCACGGTTCATTC	GCCGACGATTCAAGCTGACA
HRH3	CTCCGCACCCAGAACAACTT	GCACGATGTTGAAGACTGAGG
HRH4	GTCCCCTTGGCATTTTTAATGTC	ACATGCAGATTCCACTTCCAAA
GAPDH	CAAGGTCATCCATGACAACTTTG	GGCCATCCACAGTCTTCTGG

### Western Blotting

Nuclear protein and cytoplasmic protein were extracted from lysed primary IECs using an extraction kit (P0028; Beyotime, Shanghai, China) and phenylmethylsulfonyl fluoride (100 mM; ST506, Beyotime, Shanghai, China). Total proteins were isolated from the ileal tissues using the Minute*™* Total Protein Extraction Kit for Intestines (Invent, Eden Prairie, MN, United States). The protein lysates prepared above were electrophoresed on 10% or 6% stain-free polyacrylamide gels (Bio-Rad, Hercules, CA, United States), electrotransferred to 0.2 μm or 0.45 μm of polyvinylidene difluoride (PVDF) membranes (Millipore, Chicago, IL, United States), incubated with the primary and secondary antibodies (cell signaling technology; 1:2000) in sequence, and subjected to signal visualization using an enhanced chemiluminescent detection system (Bio-Rad, Hercules, CA, United States). Band intensities were quantified using ImageJ software (NIH, Bethesda, MD, United States). The primary antibodies used were mouse anti-CYP1A1 (Santa Cruz; sc-25304; 1:100), rabbit anti-ZO-1 (Invitrogen; PA5-85256; 1:1000), rabbit anti-E-cadherin (Cell Signaling Technology; 3195; 1:1000), rabbit anti-occludin (Invitrogen; 40-4700; 1:1000), rabbit anti-HRH4 (Bioss; bs-10165R; 1:300), rabbit anti-MLCK (Invitrogen; PA5-79716; 1:1000), rabbit anti-phospho-MLC2 (Cell Signaling Technology; 3671; 1:1000), rabbit anti-p65 (Cell Signaling Technology; 8242; 1:1000), rabbit anti-PCNA (Cell Signaling Technology; 13110; 1:1000), rabbit anti-phospho-IκBα (Cell Signaling Technology; 2859; 1:1000), and rabbit anti-β-actin (Cell Signaling Technology; 4970; 1:1000). Secondary antibodies were purchased from Cell Signaling Technology (Danvers, MA, United States).

### Statistical Analysis

The data are presented as means ± standard error (SEM) of three independent experiments. The survival rates were analyzed using the log-rank test. Multiple group comparisons were performed using a one-way analysis of variance. All statistical analyses were performed using Prism 9.0 (GraphPad Software, United States), and *P*-values < 0.05 were considered statistically significant.

## Results

### CYP1A1 Deletion Promotes Survival and Attenuates Intestinal Epithelial Dysfunction in Methicillin-Resistant *Staphylococcus aureus*-Induced Sepsis

**FIGURE 1:**
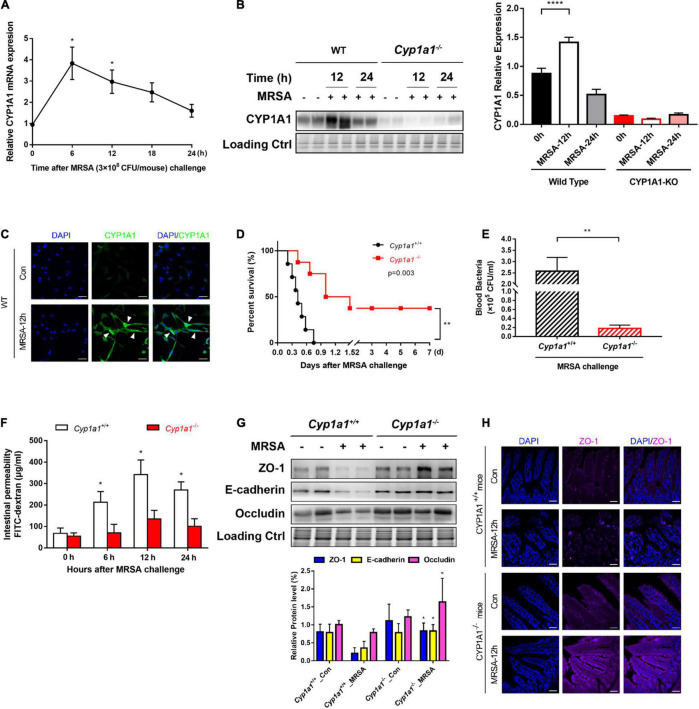
*Cyp1a1*^–/–^ mice are protected from methicillin-resistant *Staphylococcus aureus* (MRSA)-induced sepsis and intestinal barrier disruption. **(A)** mRNA levels of CYP1A1 at 12 h post-MRSA infection (p-Mi) in the ileal epithelium of wild type (WT) mice (*n* = 3–4). **(B)** Immunoblot and densitometry plots of MRSA-induced CYP1A1 levels in the ileal epithelium from CYP1A1-knockout (*Cyp1a1*^–/–^) mice (*n* = 5–6) or WT mice (*n* = 5–6). **(C)** Representative immunostaining of IECs CYP1A1 (green) from WT mouse ileum, DAPI (blue) before (Control; Con) or at 12 h p-Mi (MRSA-12 h). **(D)** Survival of *Cyp1a1*^+/+^ (*n* = 10) and *Cyp1a1*^–/–^ (*n* = 9) mice subjected to MRSA from three independent experiments. **(E)** *Cyp1a1*^+/+^ (*n* = 3) and *Cyp1a1*^–/–^ (*n* = 3) bacterial colony forming units (CFUs) in the peripheral blood were calculated at 12 h p-Mi from two independent experiments. **(F)** Intestinal permeability was measured at 12 h p-Mi from three independent experiments (*n* = 8 mice/group). **(G)** Immunoblot and densitometry plots of cell junction proteins in the ileal epithelium from two mice before or 12 h p-Mi (*Cyp1a1*^+/+^ mice, *n* = 5–6; *Cyp1a1*^–/–^ mice, *n* = 5–6). Differences were analyzed using one-way ANOVA. **(H)** Immunofluorescence staining of ileum sections of *Cyp1a1*^+/+^ mice and *Cyp1a1*^–/–^ mice for ZO-1 (purple) and DAPI (blue). Scale bar: 40 μm. Error bars, ± SEM. *P < 0.05, **P < 0.01, ****P < 0.0001.

We have previously demonstrated that CYP1A1 is overexpressed in peritoneal macrophages isolated from mice following lipopolysaccharide or *Escherichia coli* challenge (Tian et al., 2020). Similarly, the current study showed that CYP1A1 mRNA expression was altered in the ileal epithelium of MRSA-induced bacterial septic mice, increased markedly within 6 h, and then decreased (Figure 1A). Additionally, a significant increase was found in the expression of the CYP1A1 protein at 12 h post-MRSA infection (p-Mi) (Figure 1B). Consistent with these findings, the level of CYP1A1 was upregulated by confocal imaging (Figure 1C) in IECs isolated from WT mouse ileum at 12 h p-Mi relative to controls.

To further ascertain the role of Cyp1a1 in the development of MRSA-induced sepsis, we used *Cyp1a1*^–/–^ and wild-type littermates (*Cyp1a1*^+/+^ mice). *Cyp1a1*^+/+^ littermates succumbed to sepsis, whereas *Cyp1a1*^–/–^ mice showed markedly reduced mortality after MRSA challenge (hazard ratio = 4.739; 95% CI: 1.23–18.25; *P* = 0.003) (Figure 1D). Furthermore, compared to *Cyp1a1*^–/–^ mice at 12 h p-Mi, *Cyp1a1*^+/+^ mice had a significantly higher bacterial load in the peripheral blood (*P* = 0.002) (Figure 1E). Considering that the intestinal mucosal barrier is the first line of defense against invading bacteria (Turner, 2009), intestinal permeability and key cell-to-cell junctions were investigated. The expression of ZO-1, occludin, and E-cadherin was significantly increased in the ileal epithelium from *Cyp1a1*^–/–^ infected mice compared with that from *Cyp1a1*^+/+^ littermates (Figure 1G). Additionally, the effect observed at the cell junction level was similar to that at the damaged intestinal barrier, a finding consistent with the decrease in FD-4 in *Cyp1a1*^+/+^-infected mice (Figure 1F). Immunostaining also showed increased expression of ZO-1 in the ileum of *Cyp1a1*^–/–^ infected mice (Figure 1H). These data suggest that the loss of CYP1A1 renders mice less susceptible to MRSA-induced abdominal sepsis through intestinal hypo-permeability.

### Transcriptomic Changes in the Ileum Are Regulated by CYP1A1

**FIGURE 2:**
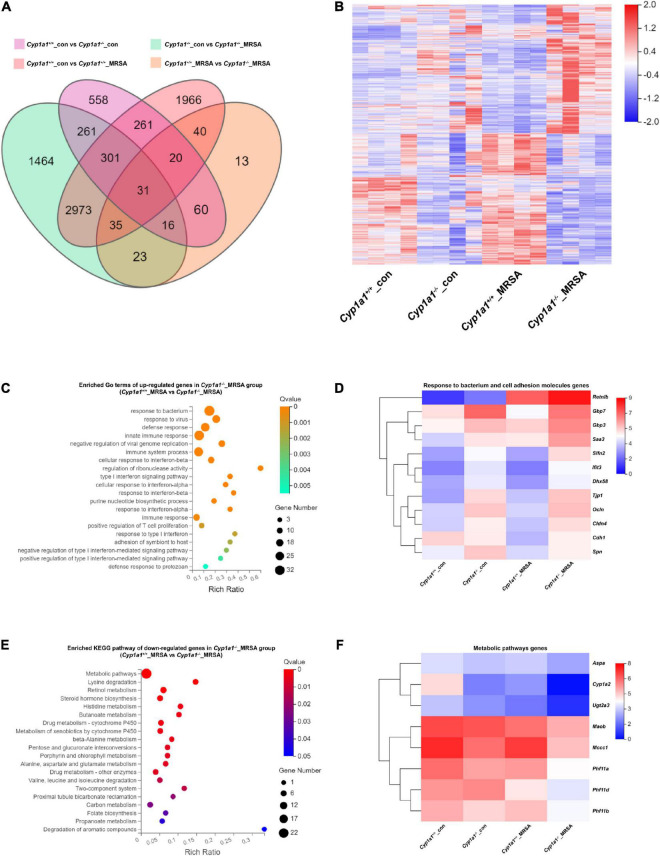
Ileum RNA profile comparison between *Cyp1a1*^+/+^ mice and *Cyp1a1*^–/–^ mice at 12 h p-Mi. **(A)** Venn diagram summarizing the expression of genes in ileum tissues from *Cyp1a1*^+/+^ mice and *Cyp1a1*^–/–^ mice with or without MRSA infection, as determined by RNA-seq data. **(B)** Heatmap of the differentially expressed genes (DEGs) in ileum tissues from mice as in **(A)**. **(C)** Functional annotation of genes by the top 20 gene ontology (GO) terms upregulated in *Cyp1a1*^–/–^ mice at 12 p-Mi. **(D)** Heatmap of the expression of the DEGs encoding bacterial response and cell adhesion molecules. **(E)** Functional annotation of genes by the top 20 Kyoto Encyclopedia of Genes and Genomes (KEGG) pathways downregulated in *Cyp1a1*^–/–^ mice at 12 h p-Mi. **(F)** Heatmap of the expression of the DEGs encoding metabolic pathways.

To investigate the potential mechanisms by which *Cyp1a1*^–/–^ mice facilitate enterocyte barrier improvements following MRSA-induced sepsis, RNA sequencing of ileal tissues from *Cyp1a1*^+/+^ and *Cyp1a1*^–/–^ mice in the absence and presence of MRSA was performed. A total of 238 DEGs were identified between the *Cyp1a1*^–/–^_MRSA and *Cyp1a1*^+/+^_MRSA groups, comprising 65 downregulated genes and 173 upregulated genes (Figures 2A,B). The GO analysis of upregulated genes in the *Cyp1a1*^–/–^_MRSA group revealed several DEGs encoding antibacterial proteins (Figures 2C,D). This finding further indicated that a series of cell adhesion molecule (CAM)-related genes, such as *Tjp1*, *Ocln*, *Cldn4*, *Cdh1*, and *Spn*, were upregulated in the *Cyp1a1*^+/+^_MRSA group (Figure 2D). Similarly, our data confirmed that both the mRNA and protein levels of cell junction markers, such as ZO-1, E-cadherin, and occludin, were increased in *Cyp1a1*^+/+^-infected mice (Figure 1G), accompanied by high mRNA levels of host defense pathway molecules, such as *Retnlb*, *Gbp7*, and *Gbp3* (data not shown). Furthermore, KEGG pathway analysis illustrated that these downregulated genes in the *Cyp1a1*^–/–^_MRSA group were primarily related to metabolic pathways, including lysine degradation, retinol metabolism, steroid hormone biosynthesis, and histidine metabolism (Figures 2E,F).

### Loss of CYP1A1 Reduces the Metabolic Shift From Lysine to Cadaverine in the Caecal Contents During Methicillin-Resistant *Staphylococcus aureus*-Induced Sepsis

**FIGURE 3:**
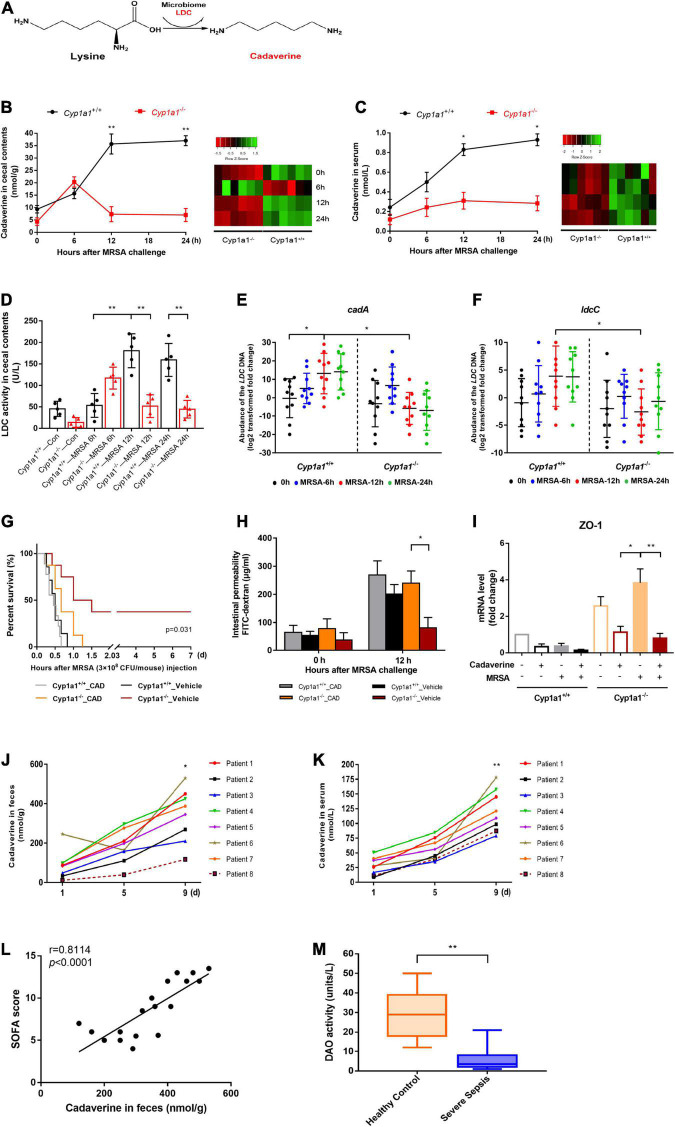
Caecal cadaverine is elevated during MRSA-induced sepsis. **(A)** Schematic diagram of cadaverine (CAD) biosynthesis from L-lysine. CAD levels in **(B)** the caecal contents and **(C)** serum (*n* = 9) were measured by UHPLC–MS following MRSA challenge. **(D)** Lysine decarboxylase (LDC) enzyme activity and RT–qPCR analysis of **(E)** *cadA* and **(F)** *ldcC* within caecal DNA samples. CAD administration to *Cyp1a1*^–/–^ mice led to **(G)** reduced survival; **(H)** a higher FITC-dextran concentration in serum, and **(I)** reduced mRNA expression of ZO-1 in the ileal epithelium, as shown by RT–qPCR. CAD levels in **(J)** feces and **(K)** serum from patients with sepsis and MRSA infection (*n* = 8). **(L)** Correlation between the fecal CAD level and sequential organ failure assessment (SOFA) score. The Spearman correlation coefficient was used for evaluation. **(M)** Serum diamine oxidase (DAO) enzyme activity from patients with sepsis and MRSA infection (*n* = 18) and healthy controls (*n* = 20). Error bars, ± SEM. *P < 0.05, **P < 0.01.

Cadaverine is produced by the decarboxylation of lysine by the LDC enzyme *via* the gut microbiome (Figure 3A). To further characterize the metabolic role of CYP1A1 during MRSA-induced sepsis, the cadaverine concentration and LDC activity were measured in the caecal contents and the serum of infected *Cyp1a1*^+/+^ and *Cyp1a1*^–/–^ mice by HPLC analysis and ELISA, respectively. Cadaverine levels and LDC activity in the caecal contents at 6 h p-Mi were mildly increased in *Cyp1a1*^+/+^ and *Cyp1a1*^–/–^ mice. However, within 24 h, the cadaverine level gradually returned to normal in *Cyp1a1*^–/–^ mice but not in *Cyp1a1*^+/+^ mice with MRSA infection (Figures 3B,D). Correspondingly, *Cyp1a1*^–/–^ mice had a lower level of cadaverine in the serum following MRSA challenge than *Cyp1a1*^+/+^ mice (Figure 3C). To further address whether intestinal cadaverine biosynthesis was modified in *Cyp1a1*^–/–^ mice, the abundance of the *LdcC* and *CadA* genes encoding LDC in fecal DNA was assessed. As expected, *Cyp1a1*^–/–^ mice had a lower abundance of the *CadA* and *LdcC* genes than *Cyp1a1*^+/+^ mice in fecal DNA at 12 h p-Mi (Figures 3E,F). Subsequently, cadaverine pretreatment (250 mg/kg) of *Cyp1a1*^–/–^ mice for 5 days impaired survival following MRSA challenge compared with that of the vehicle-treated control group; *Cyp1a1*^+/+^ mice also showed the same trend (Figure 3G). At the same time, a consistent decrease in the mRNA expression of cell junctions, such as *Zo1* and higher intestinal permeability, was observed in *Cyp1a1*^–/–^ mice treated with cadaverine (Figures 3H,I). These data demonstrated that cadaverine pretreatment could abolish the protective effects on the gut barrier function in *Cyp1a1*^–/–^ mice.

The functional relevance of cadaverine levels in fecal and serum samples from patients with sepsis and MRSA infection and healthy control individuals was assessed. Similar to the mouse results, increased cadaverine levels were consistently detected in the fecal and serum samples from critically ill patients with persistent MRSA infection (Figures 3J,K), whereas cadaverine was not detected in the healthy controls. The fecal cadaverine level was further positively correlated with the SOFA score (Figure 3L). Since biopsy samples of the ileum from critically ill patients could not be easily obtained, DAO activity in serum was used to indirectly reflect the intestinal permeability of the small intestine (Honzawa et al., 2011). Serum DAO activity in patients with sepsis and MRSA infection was significantly lower than that in the healthy control subjects (Figure 3M). Collectively, the results indicate that critically ill patients with a “leaky gut” have higher cadaverine levels.

### Decreased Cadaverine Levels Are Associated With an Altered Microbiome in *Cyp1a1*-Knockout Mice With Methicillin-Resistant *Staphylococcus aureus* Infection

**FIGURE 4:**
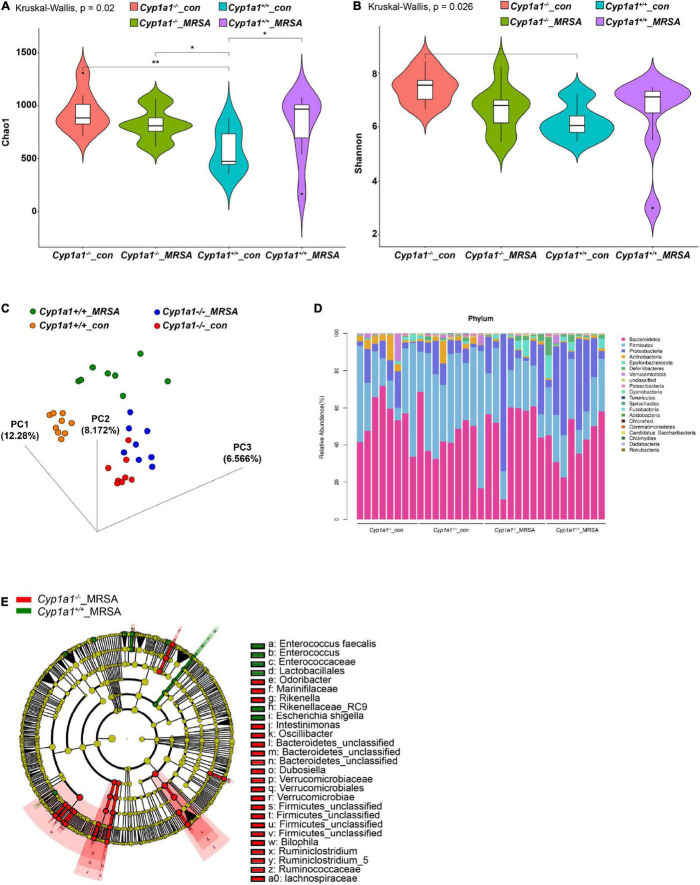
*Cyp1a1* deficiency alters the gut microbiota composition. **(A)** Chao 1 index and **(B)** Shannon index measuring bacterial richness and diversity by 16S rDNA sequencing in the caecal content of *Cyp1a1*^+/+^ mice and *Cyp1a1*^–/–^ mice with or without MRSA infection (*n* = 8–9). **(C)** The unweighted UniFrac-based 3D-PCoA showed obvious differences in the gut microbiota composition between mice in **(A)** (*n* = 8–9). **(D)** Bar chart of the relative microbiome abundance at the phylum level from mice as in **(A)**. **(E)** The cladogram based on LEfSe analysis shows bacterial taxa (class, order, family, and genus) differentially abundant in *Cyp1a1*^+/+^ mice and *Cyp1a1*^–/–^ mice with MRSA infection. Green indicates increased abundance in the *Cyp1a1*^+/+^_MRSA group; red indicates increased abundance in the *Cyp1a1*^–/–^_MRSA group. *P < 0.05, **P < 0.01.

Alterations from lysine to cadaverine profiles are closely related to the involvement of gut bacteria (Kovács et al., 2019). To correlate the specific bacterial taxa with cadaverine levels following MRSA-induced sepsis, we performed 16S ribosomal DNA sequencing and clarified the caecal microbial diversity and composition of *Cyp1a1*^+/+^ and *Cyp1a1*^–/–^ mice in the presence or absence of MRSA challenge at 24 h. The unweighted UniFrac-based 3D-PCoA of all caecal microbiota revealed distinct segregation among these groups (Figure 4C, Supplementary Table 3), although no significant difference was found in the Shannon diversity of the gut flora between MRSA groups (Figure 4B, Supplementary Table 1). However, the bacterial richness was enormously decreased in *Cyp1a1*^+/+^ mice compared with that in *Cyp1a1*^–/–^ mice under basal conditions (Figure 4A, Supplementary Table 1). In particular, extreme dysbiosis was found at the phylum level namely, an increase in *Proteobacteria* and a loss of *Bacteroidetes* in *Cyp1a1*^+/+^ mice compared to those in *Cyp1a1*^–/–^ mice at 24 h p-Mi (Figure 4D). Notably, *Cyp1a1*^–/–^ mice demonstrated significantly fewer cadaverine-producing taxa than *Cyp1a1*^+/+^ mice, as shown by the LEfSe analysis presented by a cladogram. The main differences between the *Cyp1a1*^+/+^_MRSA and *Cyp1a1*^–/–^_MRSA groups were a reduction in the abundance of *E. faecalis* (phylum *Firmicutes*), *Escherichia shigella* (phylum *Proteobacteria*), and *Rikenellaceae* (phylum *Bacteroidetes*) along with an increase in *Odoribacter* (phylum *Bacteroidetes*), *Marinifilaceae* (phylum *Bacteroidetes*), *Rikenella* (phylum *Bacteroidetes*), *Intestinimonas* (phylum *Firmicutes*), *Oscillibacter* (phylum *Firmicutes*), *Verrucomicrobiae* (phylum *Verrucomicrobia*), *Ruminococcaceae* (phylum *Verrucomicrobia*), and *Lachnospiraceae* (phylum *Firmicutes*) (Figure 4E, Supplementary Table 2). These results demonstrate that CYP1A1 deficiency plays an essential role in remodeling gut microbiota composition and reducing the abundance of opportunistic pathogens and flora imbalance following MRSA challenge.

### Antibiotic Administration-Induced Intestinal Dysbacteriosis Disrupts Intestinal Barrier Integrity in *Cyp1a1*-Knockout Mice

To further validate whether the protective effect of *Cyp1a1*^–/–^ mice is partly mediated by the gut microbiome, we administered a broad-spectrum of antibiotic cocktail (ABX) containing vancomycin (0.5 g/L; Sigma), ampicillin (1 g/L; Sigma), neomycin (1 g/L; Sigma), and metronidazole (1 g/L; Sigma) to *Cyp1a1*^+/+^ mice and *Cyp1a1*^–/–^ mice twice a day for 2 weeks before abdominal sepsis modeling. Importantly, the survival benefit of *Cyp1a1*^–/–^ mice was significantly lost after ABX-induced intestinal dysbacteriosis, indicating that the intact gut microbiota in *Cyp1a1*^–/–^ mice are required to combat MRSA-induced abdominal sepsis (Supplementary Figure 1A). Additionally, the level of cadaverine in the caecal contents was markedly increased in ABX-treated *Cyp1a1*^+/+^ mice and *Cyp1a1*^–/–^ mice compared to that in control mice that were not administered ABX (Supplementary Figure 1B). This finding agrees with the expectation that ABX perturbs gut microbiota and leads to metabolic disorders (de Jong et al., 2020). Furthermore, immunostaining analysis of ileal tissue confirmed the decrease in ZO-1 expression in *Cyp1a1*^–/–^ mice that received ABX when subjected to MRSA infection (Supplementary Figure 1C). Taken together, these results further support that the ABX-driven gut microbiome perturbation to *Cyp1a1*^–/–^ mice negates the survival benefit and disrupts intestinal barrier integrity after MRSA-induced sepsis.

### *Enterococcus faecalis* Counteracts the Original Intestinal Barrier Protection in *Cyp1a1*-Knockout Mice With Methicillin-Resistant *Staphylococcus aureus* Infection

**FIGURE 5:**
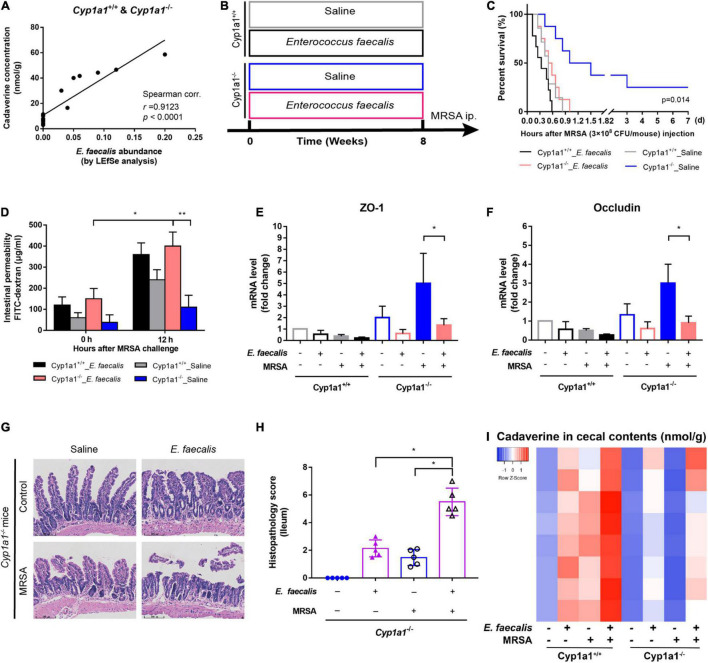
Cadaverine-producing *Enterococcus faecalis* causes gut barrier disruption and increased mortality. **(A)** Correlation analysis between the cadaverine (CAD) level and paired *E. faecalis* abundance in the caecal contents from *Cyp1a1*^+/+^ mice and *Cyp1a1*^–/–^ mice with MRSA infection. **(B)** *Cyp1a1*^+/+^ mice and *Cyp1a1*^–/–^ mice were treated daily with saline or *E. faecalis* (4 × 10^7^ CFU) by oral gavage for 8 weeks. *E. faecalis* treatment of *Cyp1a1*^–/–^ mice led to **(C)** increased mortality, **(D)** higher fluorescein isothiocyanate (FITC)-dextran concentration in serum, and reduced mRNA levels of **(E)** zonula occludens (ZO)-1 and **(F)** occludin in the ileum epithelium. **(G)** Representative H&E staining in the ileum from *Cyp1a1*^–/–^ mice with or without *E. faecalis* treatment. Scale bar, 100 μm. **(H)** Histological scores of the ileum in each group (*n* = 4–5). **(I)** Heatmap of the caecal contents from each group. Error bars, ±SEM. *P < 0.05, **P < 0.01.

*Enterococcus* is one of the most cadaverine-producing commensal bacteria in the gut. Thus, its abnormal colonization can disrupt the intestinal microecology due to the accumulation of harmful microbial products (Zitvogel and Kroemer, 2019). As expected, the abundance of *E. faecalis* was positively correlated with the cadaverine concentration in *Cyp1a1*^+/+^ and *Cyp1a1*^–/–^ mouse intestines (*r* = 0.9123, *p* < 0.0001), suggesting the role of *E. faecalis* in the shift from lysine production toward cadaverine production (Figure 5A and Supplementary Figures 2A,B). Since the data pointed to the abundance of *E. faecalis* in *Cyp1a1*^+/+^ mice, which succumbed to sepsis, we investigated the direct influence of *E. faecalis* treatment on gut barrier integrity. Therefore, we treated *Cyp1a1*^+/+^ mice and *Cyp1a1*^–/–^ mice daily with a commercial strain of *E. faecalis* (4 × 10^7^ CFU/mouse) or saline for 10 weeks by oral gavage before MRSA challenge (Figure 5B). All the animals receiving *E. faecalis* died within 24 h p-Mi, whereas the survival of *Cyp1a1*^–/–^ mice not receiving *E. faecalis* was extended (Figure 5C). Furthermore, *E. faecalis* treatment significantly increased intestinal permeability, accompanied by a reduction in *Zo1* and *Ocln* mRNA expression in the ileal epithelium and increased histopathology scores for the ileum in both *Cyp1a1*^+/+^ mice and *Cyp1a1*^–/–^ mice following MRSA challenge (Figures 5D–H). *E. faecalis* treatment also effectively increased the cadaverine levels in the caecal contents from *Cyp1a1*^+/+^ and *Cyp1a1*^–/–^ mice (Figure 5I). Thus, *E. faecalis* displays a disruptive genotype that causes gut leakiness in mice.

### Microbiota-Derived Cadaverine Induces Enterocyte Junction Disruption by Activating the HRH4/NF-κB/Myosin Light Chain Kinase Signaling Pathway

**FIGURE 6:**
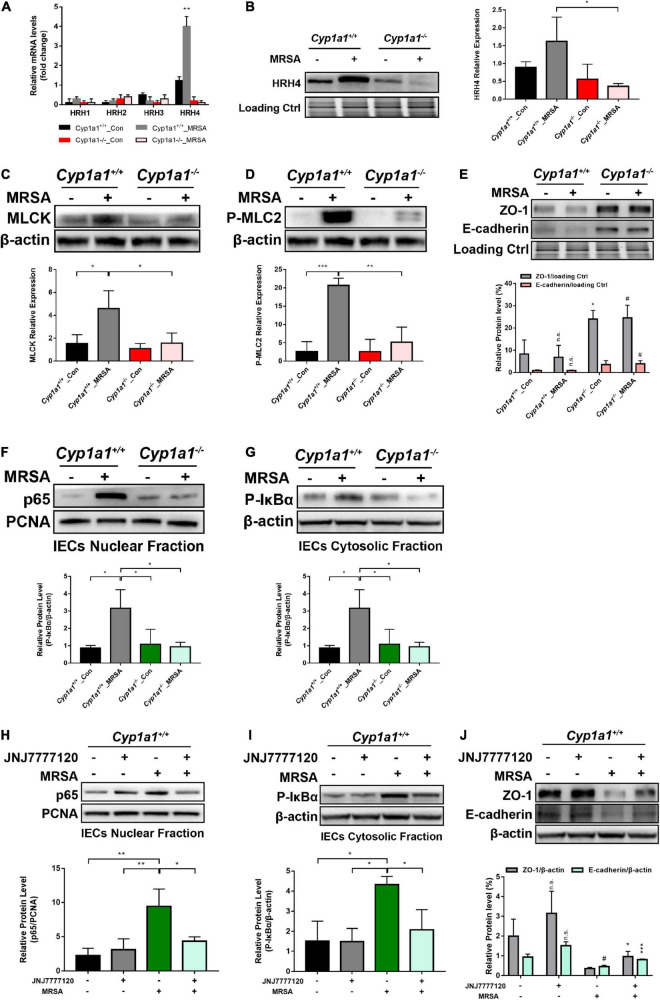
Microbiota-derived CAD induces gut barrier disruption through HRH4/NF-κB/MLCK activation. **(A)** Relative mRNA expression of HRH1, HRH2, HRH3, and HRH4 in the ileal epithelium from each group (*n* = 3–4). **(B)** Immunoblot and densitometry plots of the MRSA-induced HRH4 levels in the ileal epithelium from each group (*n* = 5–6). **(C,D)** Immunoblot and densitometry plots of MLCK **(C)** and P-MLC2 **(D)** in whole-cell lysates of IECs from each group (*n* = 5–6). **(E)** Immunoblot and densitometry plots of tight junction proteins in the ileal epithelium from each group (*n* = 6–7). **(F,G)** Immunoblot and densitometry plots of the p65 levels in the nuclear extracts **(F)** and those of P-IκBα levels in the cytosolic extracts **(G)** of IECs from two mice before or at 12 h p-Mi (*Cyp1a1*^+/+^ mice, *n* = 6–7; *Cyp1a1*^–/–^ mice, *n* = 6–7). PCNA is a nuclear loading control. **(H–J)** Immunoblot and densitometry plots of the p65 levels in the nuclear extracts **(H)**, P-IκBα levels in the cytosolic extracts **(I)**, and tight junction protein levels in whole-cell lysates **(J)** of IECs from each group (*n* = 6–7). Error bars, ± SEM. Differences were analyzed using one-way ANOVA. *P < 0.05, **P < 0.01, ***P < 0.001, n.s. not significant. ^#^Comparison between *Cyp1a1*^+/+^_MRSA and *Cyp1a1*^–/–^_MRSA groups or between JNJ7777120^–^MRSA^+^ and JNJ7777120^+^MRSA^+^ groups. ^#^*P* < 0.05.

Based on the functional screening of small molecules, cadaverine was recognized as a bacterial metabolite likely responsible for histamine receptor (HRH) family member HRH4 agonism (Colosimo et al., 2019). Consistently, only HRH4 was markedly increased in the ileal epithelium of *Cyp1a1*^+/+^ mice compared with that in *Cyp1a1*^–/–^ mice following MRSA infection, with no significant changes in other receptors, such as HRH1, HRH2, and HRH3 (Figures 6A,B). Intestinal barrier loss reflects the increased permeability of the cell-to-cell tight junction due to myosin light chain kinase (MLCK) activation and myosin II regulatory light chain (MLC2) phosphorylation (Otani and Furuse, 2020). We observed increased MLCK and p-MLC2 protein expression in IECs from *Cyp1a1*^+/+^ mice at 12 h p-Mi, accompanied by a substantial decrease in the expression of ZO-1 and E-cadherin. In contrast, decreased MLCK and phospho-MLC2 expression and a concomitant increased ZO-1 and E-cadherin expression were observed in IECs from *Cyp1a1*^–/–^ mice at 12 h p-Mi (Figures 6C–E). NF-κB p65 can bind to the MLCK promoter region to cause MLCK-mediated MLC phosphorylation (Lawrence, 2009; Han et al., 2017), and participates in histamine-mediated signaling (Huang et al., 2011). Therefore, we evaluated the activation of NF-κB and found that nuclear expression of p65 and cytoplasmic expression of phospho-IκBα were markedly increased in IECs from *Cyp1a1*^+/+^-infected mice, whereas *Cyp1a1*^–/–^-infected mice showed a low level of p65 translocation to the nucleus (Figures 6F,G). Furthermore, the effect of the HRH4 antagonist JNJ7777120 (150 mg/kg) on cadaverine-induced junctional protein disruption was examined. This cadaverine-induced decrease in ZO-1 and E-cadherin in the ileal epithelium was significantly reversed by JNJ7777120 in *Cyp1a1*^+/+^-infected mice (Figure 6J). HRH4 signaling blockade by JNJ7777120 also reduced the nuclear translocation of NF-κB p65 and cytosolic level of phospho-IκBα in the IECs of *Cyp1a1*^+/+^-infected mice (Figures 6H,I). Collectively, these results indicate that the binding of cadaverine to HRH4 induces gut barrier dysfunction by activating NF-κB/MLCK signaling.

### CYP1A1 Deficiency Ameliorates Gut Barrier Dysfunction Induced by Commensal Bacteria in an Aryl Hydrocarbon Receptor-Independent Manner

**FIGURE 7:**
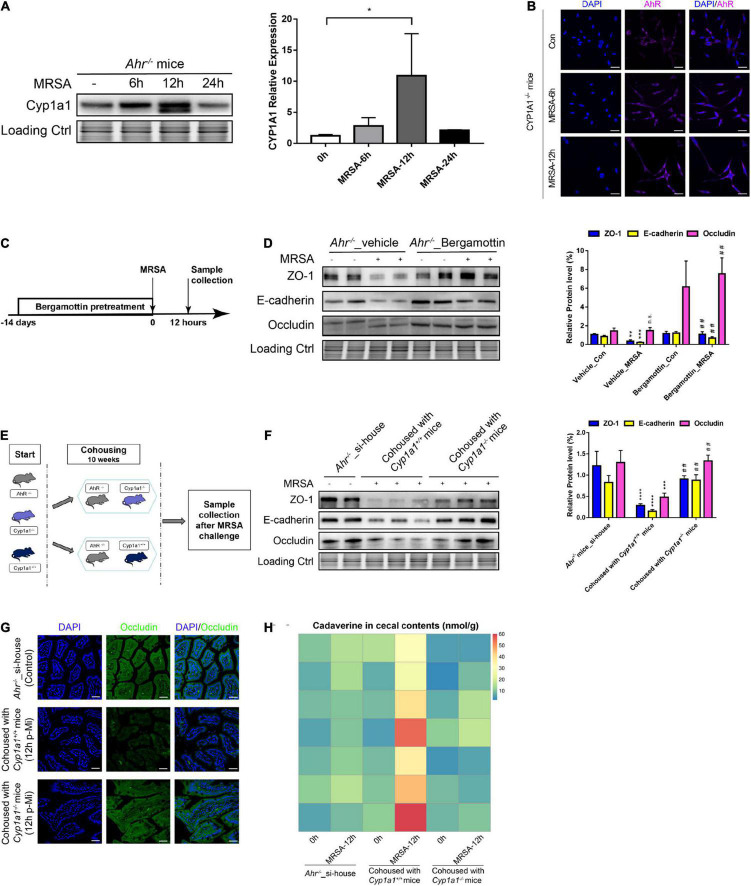
Gut barrier function induced by commensal bacteria in an AHR-independent manner. **(A)** Immunoblot and densitometry plots of MRSA-induced CYP1A1 levels in the ileal epithelium from *Ahr*^–/–^ mice (*n* = 5–6). **(B)** Representative immunostaining of IEC AHR (purple) and DAPI (blue) from *Cyp1a1*^–/–^ mouse ileum before (Control; Con) or 6 h (MRSA-6h) and 12 h p-Mi (MRSA-12h). **(C)** Experimental design. *Ahr*^–/–^ mice were fed bergamottin for 14 days before the MRSA challenge. Ileum mucosa collection at 12 h p-Mi for immunoblotting. **(D)** Immunoblot and densitometry plots of cell junction proteins in the ileal epithelium from *Ahr*^–/–^_Vehicle mice (*n* = 7–8) or *Ahr*^–/–^_Bergamottin mice (*n* = 7–8). **(E)** Experimental design. *Ahr*^–/–^ mice were cohoused with *Cyp1a1*^+/+^ mice and *Cyp1a1*^–/–^ mice before MRSA challenge for 10 weeks. Sample collection at 12 h p-Mi. **(F)** Immunoblot and densitometry plots of cell junction proteins in the ileal epithelium from *Ahr*^–/–^_si-house mice (*n* = 7–8), *Ahr*^–/–^ mice cohoused with *Cyp1a1*^+/+^ mice (*n* = 8–10) or *Ahr*^–/–^ mice cohoused with *Cyp1a1*^–/–^ mice (*n* = 8–10). **(G)** Immunofluorescence staining of ileum sections for occludin (green) and DAPI (blue) from each group. Scale bar: 40 μm. **(H)** Heatmap of the caecal contents from each group. Error bars, ±SEM. *P < 0.05. si-house, only *Ahr*^–/–^ mice were housed in a cage; cohouse, *Ahr*^–/–^ mice were housed in a cage with *Cyp1a1*^+/+^ mice or *Cyp1a1*^–/–^ mice separately. Differences were analyzed using one-way ANOVA. *P < 0.05, **P < 0.01, ***P < 0.001, ****P < 0.0001, n.s., not significant. ^##^Comparison between *Ahr*^–/–^_Vehicle-MRSA and *Ahr*^–/–^_Bergamottin-MRSA groups, or *Ahr*^–/–^ mice cohoused with *Cyp1a1*^+/+^ mice and *Ahr*^–/–^ mice cohoused with *Cyp1a1*^–/–^ mice. ^##^*P* < 0.01.

Since feedback control of AHR-CYP1A1 signaling is required to regulate intestinal immune functions (Schiering et al., 2017), we analyzed the expression of CYP1A1 and AHR in IECs from AHR KO and CYP1A1 KO mice. The increased level of CYP1A1 was confirmed by western blot analysis in *Ahr*^–/–^ mouse IECs following MRSA challenge as early as 6 h and was restored to normal levels at 24 h p-Mi (Figure 7A). Additionally, CYP1A1 deletion did not show a prominent effect on the nuclear translocation of AHR in IECs but was slightly accumulated in the cytoplasm after MRSA infection as demonstrated by immunofluorescence assay (Figure 7B). Furthermore, pathway analysis of our RNA-seq data did not detect AHR-related pathways in *Cyp1a1*^–/–^ mice with MRSA infection (Figure 2E). Previously, we found that using a CYP1A1 inhibitor improved the survival and bacterial clearance in septic mice (Tian et al., 2020). Notably, we also found that the key enterocyte junction proteins, such as ZO-1, E-cadherin, and occludin, were markedly increased in the ileal epithelium of *Ahr*^–/–^ mice after treatment with bergamottin, a competitive inhibitor of CYP1A1 (Figures 7C,D).

Since the gut microbiota confers gut barrier dysfunction by metabolizing lysine to cadaverine in the gut of *Cyp1a1*^+/+^ mice under MRSA infection, we cohoused *Ahr*^–/–^ mice with *Cyp1a1*^+/+^ mice and *Cyp1a1*^–/–^ mice to investigate whether direct gut microbiota-mediated signaling occurs in a non-AHR dependent manner following MRSA challenge (Figure 7E). As shown in Figure 7F, increased expression of ZO-1, E-cadherin, and occludin in the ileal epithelium was detected in *Ahr*^–/–^ mice after cohousing with *Cyp1a1*^–/–^ mice for 10 weeks but not after cohousing with *Cyp1a1*^+/+^ mice. Additionally, immunostaining revealed similar changes in occludin expression in the ileum of *Ahr*^–/–^ mice cohoused with *Cyp1a1*^–/–^ mice, suggesting an improved epithelial barrier after floral transfer from *Cyp1a1*^–/–^ mice (Figure 7G). Therefore, we asked whether AHR-KO mice at 10 weeks post-cohousing contributed to the deregulation of lysine degradation. The increased level of cadaverine at 12 h p-Mi in *Ahr*^–/–^ mice after cohousing with *Cyp1a1*^+/+^ mice but not with *Cyp1a1*^–/–^ mice suggests that unique bacterial taxa in *Cyp1a1*^–/–^ mice induce events that promote cell-to-cell junctions of enterocytes (Figure 7H). These data highlight that although AHR plays a key role in safeguarding the barrier function, the microbiome-metabolite axis may govern the effect on the gut barrier in *Cyp1a1*^–/–^ mice independent of AHR.

## Discussion

Gut barrier dysfunction is considered both a result and cause of sepsis development (Haussner et al., 2019). For example, sepsis changes from the symbiotic intestinal microenvironment to a dysbiotic environment with a predominance of pathogenic bacteria and harmful metabolites (Fay et al., 2017; Adelman et al., 2020). Decreased production of beneficial microbial products directly interacts with IECs and impacts host health (Ghosh et al., 2021). CYP1A1 plays a critical role in regulating the inflammatory response and phagocytosis in individuals with sepsis and may modulate the gut microbiota (Obata et al., 2020; Tian et al., 2020). A recent study showed that dietary phytochemicals strengthen the epithelial barrier by inhibiting CYP1A1 and causing alterations in the microbiome composition (Rannug, 2020). The research covered in this report showed that genetic ablation of CYP1A1 contributes to intestinal barrier protection against MRSA-induced sepsis by shifting the microbiota composition to decrease cadaverine metabolite levels in a non-AHR-dependent manner. Additionally, the above gut microbiota-mediated protective effects are negated in the presence of antibiotics. Furthermore, oral supplementation with cadaverine or a commercial strain of *E. faecalis* counteracts the original protection conferred to *Cyp1a1* KO mice with MRSA infection.

Emerging evidence suggests that polymorphisms in the CYP1A1 gene partly contribute to the increased vulnerability to CAP and NP (Salnikova et al., 2013; Zhao et al., 2017). However, the detailed effect of CYP1A1 on intestinal barrier function in sepsis caused by drug-resistant bacteria remains to be explored. Constitutive CYP1A1 overexpression results in reduced peristaltic activity in the colon and increased susceptibility to enteric infection (Schiering et al., 2017; Obata et al., 2020). Motivated by these discoveries, we analyzed the transcriptome of ileal sections by RNA-seq and identified a series of downregulated genes in the *Cyp1a1*^–/–^_MRSA group that were primarily enriched in metabolic pathways. Among these pathways, we focused on lysine degradation, whose expression was strongly downregulated by CYP1A1 deficiency. Cadaverine production from lysine degradation by commensal bacteria requires the specific bacterial enzyme, LDC. Therefore, the limiting factor may not be the substrate lysine but the presence of bacteria that produce cadaverine, particularly *Enterococcus*, *Streptococcus*, and *Escherichia/Shigella* (Mu et al., 2016). Notably, we described for the first time the gut microbiota in Cyp1a1 KO mice under both basal conditions and MRSA-induced sepsis conditions, confirming the previously held notion that inhibiting CYP1A1 contributes to maintaining homeostasis by changing microbiota-associated cascade signaling (Rannug, 2020).

Although numerous studies have performed phenotypic characterization of bacterial aetiologies, less is known about gut microbiome changes after MRSA-induced sepsis. The main differences we observed after MRSA-induced sepsis in this study were an increased abundance of *E. faecalis* and *E. shigella* in *Cyp1a1*^+/+^ mice, but these changes were not observed in *Cyp1a1*^–/–^ mice. These findings in *Cyp1a1*^+/+^ mice are consistent with the evidence supporting that gut microbiota alteration in patients with sepsis is characterized by significant increases in the genus, *Enterococcus* (Wilmore et al., 2018; Liu et al., 2021). In particular, enteric dysbiosis dominated by *Enterococcus* has been reported to be a high-risk factor for mortality among patients with sepsis (Adelman et al., 2020; Agudelo-Ochoa et al., 2020). Furthermore, the reduction in helpful metabolites, such as butyrate, a health-promoting effector mediating anti-inflammatory signals in the intestine, has been specifically associated with the presence of *Enterococcus* species (Wolff et al., 2018; Agudelo-Ochoa et al., 2020). These data suggest that opportunistic pathogens are increased while health-promoting bacteria and metabolites are reduced following MRSA infection. The loss of CYP1A1, which protects against bacterial sepsis, limits the abundance of *E. faecalis* and increases taxa associated with metabolic health, such as *Intestinimonas butyriciproducens*, under MRSA challenge. Although the precise mechanisms by which host genetics influence the composition of gut microbes must be fully elucidated, our data suggest a dynamic host–microbiome relationship within CYP1A1 and specific taxa. Additional work on genome-wide association is required to understand how CYP1A1 deficiency modulates the gut microbiota against MRSA-induced sepsis.

Additionally, mice fed with the same diet have different microbial metabolites, likely explaining interindividual differences in the gut microbiome (Smits et al., 2016). Using UHPLC–MS analysis, we found that the gut microbial metabolite cadaverine was accumulated in *Cyp1a1*^+/+^ mice with MRSA infection due to the abundance of *E. faecalis*. Genetic deletion of CYP1A1 not only suppressed *E. faecalis* but also reduced the level of cadaverine after MRSA challenge. Cadaverine was originally identified as a death-associated odor (Hussain et al., 2013) and a harmful metabolite in the gut. The supplementation of cadaverine in *Cyp1a1*^–/–^ mice with MRSA increased mortality and disrupted cell junctions, suggesting that the high level of cadaverine was harmful to the maintenance of host health. In parallel, this phenomenon did not appear to be limited to mice, as higher cadaverine levels were also detected in fecal and serum samples from patients with sepsis and persistent MRSA infection but absent in healthy volunteers. Additionally, serum DAO activity in patients with sepsis and MRSA infection was significantly lower than that in healthy controls, indicating that critically ill patients with gut leakiness had higher cadaverine levels. Furthermore, the elevated cadaverine level in feces was positively correlated with the SOFA score in these patients. Despite the limited number of participants, our findings support the view that patients with traumatic brain injuries, severe liver insufficiency, and Crohn’s disease have higher cadaverine levels (Pelletti et al., 2019; Diederen et al., 2020).

Excess antibiotic use in critically ill patients can also lead to severe microbial dysbiosis due to the combined effect of detrimental metabolite production and intestinal microenvironment disturbance. Recently, cadaverine had a dose-dependent cytotoxic effect toward HT29 intestinal cells in *in vitro* cultures (Del Rio et al., 2019); however, the specific mechanism underlying this effect remains unknown. Further study revealed that cadaverine might be responsible for HRH4 agonism (Colosimo et al., 2019). Consistently, a higher level of cadaverine in *Cyp1a1*^+/+^ infected mice had a high affinity for HRH4 compared with that in *Cyp1a1*^–/–^ mice. Although few studies have described the effects of cadaverine and its receptors on the epithelial function, the efficacy of antihistamines in the airway epithelial barrier dysfunction might reflect HRH4-mediated actions (De Benedetto et al., 2015). In addition to the activation of the MAPK and JAK/STAT signaling pathways by HRH4 (Morse et al., 2001; Horr et al., 2006; Michel et al., 2008), we observed that the cadaverine-HRH4 interaction activated classic NF-κB signaling, which facilitated MLCK-mediated major TJ protein (ZO-1 and occludin) and AJ protein (E-cadherin) disruption in *Cyp1a1*^+/+^ mice with MRSA infection. Thus, the cadaverine-induced signaling cascade is complex, and the heterogeneity in histamine receptors leads to the activation of multiple downstream signaling pathways (De Benedetto et al., 2015). The potential ability to suppress CYP1A1 or inhibit intestinal cadaverine-HRH4 signaling to enhance IEC junctions, or a combination of these two strategies, warrants further study to develop more effective sepsis treatment.

Interestingly, another novel finding from this study was that CYP1A1 KO-induced alterations in the intestinal flora and metabolites reduced the disruption of IEC junctions in mice, an effect that was transmissible by cohousing with *Ahr*^–/–^ mice. The AHR plays a protective role in regulating homeostatic processes at barrier sites, particularly in the intestinal microenvironment (Stockinger et al., 2021). Accordingly, AHR-deficient mice exhibit enhanced susceptibility to severe colitis and *Citrobacter rodentium* infection (Li et al., 2011; Rannug, 2020). Although genetic deletion of CYP1A1 delays natural ligand metabolism, resulting in increased AHR signaling (Schiering et al., 2017), the nuclear translocation of AHR was not induced in the IECs of ileum from *Cyp1a1*^–/–^ mice under MRSA challenge in the present study. Two potential reasons could explain this finding: (1) this action may be enabled by the AHR non-genomic pathway rather than the classic genomic pathway, which is primarily characterized by AHR nuclear translocation, or (2) IECs act as sensors for AHR ligands and then transmit signals across the epithelial layer to mucosal immune cells (Schiering et al., 2017; Stockinger et al., 2021). In the absence of gut bacteria that produce AHR ligands or a lack of endogenous ligands, a quasi-AHR-deficient state could exist persistently unless exogenous dietary supplements are used (Pernomian et al., 2020). We cohoused AHR-KO and CYP1A1-KO mice to directly explore the impact of unique metabolites generated by intestinal bacteria on gut barrier function, regardless of host genetic defects in AHR. Specific bacterial taxa exchanged horizontally from *Cyp1a1*^+/+^ mice produced a higher level of cadaverine with increased intestinal permeability and gut barrier disruption after MRSA challenge, whereas these effects were significantly reversed in *Ahr*^–/–^ mice cohoused with *Cyp1a1*^–/–^ mice. Consistent with human studies, the degree of horizontal transmission predicted bacterial genera with pathogenic representatives responsible for enteric infection (Moeller et al., 2018). Furthermore, these results provide indications for the future: (1) Donors need equal and careful screening before clinical fecal microbiota transplantation (FMT) in sepsis. Although FMT has been applied in patients with sepsis to correct dysbiosis, recent studies have reported that *E. coli* infections acquired *via* FMT can cause death (DeFilipp et al., 2019). Given the gut leakiness observed in *Ahr*^–/–^ mice after cohousing with *Cyp1a1*^+/+^ mice, genetic polymorphisms, such as CYP1A1, may need to be examined in donors before FMT. (2) Although the present study demonstrates novel AHR-independent effects on the microbial-metabolite axis, it does not rule out a significant role of AHR in coordinating the intestinal epithelium and mucosal immune system *via* an unidentified mechanism. Therefore, further study will be required to dissect the effect of host–microbe interactions on the mucosal barrier using specific deletion of AHR and CYP1A1 in the intestinal epithelial cells from transgenic mice.

However, this study has limitations that should be mentioned. We only investigated the impact of the whole-body deletion of CYP1A1 on microbiota-mediated metabolic signaling during abdominal sepsis-induced intestinal epithelial disruption. Since enteric stromal and immune cells in the gut also express CYP1A1, we could not rule out the possibility that these cells might also contribute to the host–microbiome crosstalk. Therefore, a mouse model of selective CYP1A1 deletion in IECs will be needed to fully demonstrate the role of the CYP1A1-microbiota metabolic axis in sepsis-induced epithelial barrier dysfunction. Additionally, primary IECs from patients with sepsis are difficult to obtain in clinical practice, and the usage of human intestinal organoid culture model seems to be a better experimental solution in follow-up studies. Although the present study suggests that host CYP1A1 plays a direct or indirect role in microbiota-mediated cadaverine metabolism during MRSA-induced sepsis, other mechanisms may exist, with or without the involvement of the microbiome. Finally, due to the fragile gut microbial equilibrium in patients with severe sepsis, further studies are needed to clarify whether a similar mechanism participates in patients with sepsis and MRSA infection.

## Conclusion

This study reveals an unexpected mechanism of host CYP1A1 in microbiota-mediated cadaverine metabolism during MRSA-induced abdominal sepsis, through which host–microbiota metabolic axes directly affect enterocyte junction disruption in an AHR-independent manner. Therefore, suppressing CYP1A1 or blocking intestinal cadaverine-HRH4 signaling by modulating host–microbiome crosstalk represents a promising strategy to maintain gut barrier homeostasis to treat abdominal sepsis.

## Data Availability Statement

The datasets presented in this study can be found in online repositories. The names of the repository/repositories and accession number(s) can be found here: Accession to cite for transcriptomics SRA data: PRJNA805025. Accession to cite for 16S rDNA SRA data: PRJNA804256.

## Ethics Statement

The studies involving human participants were reviewed and approved by Ethics Committee of Army Medical Center of PLA. The patients/participants provided their written informed consent to participate in this study. The animal study was reviewed and approved by Laboratory Animal Welfare and Ethics Committee of Third Military Medical University.

## Author Contributions

HL and XM conceived and designed the study. XM, HJ, and XC conducted most of the experiments and wrote the manuscript. WD and WT helped with protein extraction and western blotting analysis. GL and JZ assisted with cellular immunofluorescence. XuY provided three models of transgenic mice. FW and WL helped with animal experiments. XiY assisted with clinical data collection. HL interpreted the data and supervised the work. All authors read and approved the final manuscript.

## Conflict of Interest

The authors declare that the research was conducted in the absence of any commercial or financial relationships that could be construed as a potential conflict of interest.

## Publisher’s Note

All claims expressed in this article are solely those of the authors and do not necessarily represent those of their affiliated organizations, or those of the publisher, the editors and the reviewers. Any product that may be evaluated in this article, or claim that may be made by its manufacturer, is not guaranteed or endorsed by the publisher.

## Footnotes

1
http://www.geneontology.org/

2
https://www.kegg.jp/

3
https://en.wikipedia.org/wiki/Hypergeometric_distribution

4
https://bioconductor.org/packages/release/bioc/html/qvalue.html

5
http://report.bgi.com

## Supplementary Material

Supplementary Figure 1 Antibiotic administration reverses the protection of intestinal barrier integrity in *Cyp1a1*-knockout mice. **(A)** Survival of *Cyp1a1*^+/+^ (*n* = 8) and *Cyp1a1*^–/–^ (*n* = 8) mice in the presence or absence of antibiotic cocktail (ABX) and subjected to MRSA from two independent experiments (**P* < 0.05; log-rank analysis). **(B)** Heatmap of the caecal contents from each group. **(C)** Immunofluorescence staining of ileum sections for ZO-1 (green) and DAPI (blue) from each group (scale bar: 50 μm); white boxes indicate the magnified areas (scale bar: 20 μm).

Supplementary Figure 2 Spearman’s correlation analysis. Correlation analysis between the CAD level and paired *E. faecalis* abundance in the caecal contents from *Cyp1a1*^+/+^ mice **(A)** and *Cyp1a1*^–/–^ mice **(B)** with MRSA infection.

Supplementary Table 1 Alpha diversity between *Cyp1a1*^+/+^ and *Cyp1a1*^–/–^ mice in the presence or absence of MRSA challenge at 24 h.

Supplementary Table 2 Features profiling between *Cyp1a1*^+/+^ and *Cyp1a1*^–/–^ mice in the presence or absence of MRSA challenge at 24 h.

Supplementary Table 3 The abundance-unweighted Unifrac distance matrix between *Cyp1a1*^+/+^ and *Cyp1a1*^–/–^ mice in the presence or absence of MRSA challenge at 24 h.

## Abbreviations

ABX: antibiotic cocktail
AHR: aryl hydrocarbon receptor
CAMs: cell adhesion molecules
CAP: community-acquired pneumonia
CFU: colony-forming unit
CYP1A1: cytochrome P450family 1, subfamily A, polypeptide 1
DAO: diamine oxidase
DEGs: differentially expressed genes
ELISA: enzyme-linked immunosorbent assay
FITC: fluorescein isothiocyanate
FMT: fecal microbiota transplantation
GAPDH: glyceraldehyde-3-phosphate dehydrogenase
GO: gene ontology
H&E: hematoxylin and eosin
HRH: histamine receptor
IECs: intestinal epithelial cells
KEGG: Kyoto encyclopedia of genes and genomes
KO: knockout
LDC: lysine decarboxylase
MLCK: myosin light chain kinase
MLC2: myosin II regulatory light chain
MODS: multiple organ dysfunction syndrome
MRSA: methicillin-resistant *Staphylococcus aureus*
NP: nosocomial pneumonia
p-Mi: post-MRSA infection
QC: quality control
qPCR: quantitative polymerase chain reaction
SEM: standard error
SOFA: sequential organ failure assessment
UHPLC: ultra-high-performance liquid chromatography
WT: wild type
ZOs: zonula occludens.
