# Supplementary material for: The Host CYP1A1-Microbiota Metabolic Axis Promotes Gut Barrier Disruption in Methicillin-Resistant Staphylococcus aureus-Induced Abdominal Sepsis
Source: Front Microbiol. 2022 Apr 27;13:802409. doi: 10.3389/fmicb.2022.802409 (PMC9093654; doi:10.3389/fmicb.2022.802409)

Supplementary Fig. 1 Antibiotic administration reverses the protection of intestinal barrier integrity in Cyp1a1-knockout mice.

A Survival of *Cyp1a1*$^{+/+}$ (n = 8) and *Cyp1a1*$^{-/-}$ (n = 8) mice in the presence or absence of antibiotic cocktail (ABX) and subjected to MRSA from two independent experiments (**P* < 0.05; log-rank analysis). **B** Heatmap of the caecal contents from each group. **C** Immunofluorescence staining of ileum sections for ZO-1 (green) and DAPI (blue) from each group (scale bar: 50 μm); white boxes indicate the magnified areas (scale bar: 20 μm).

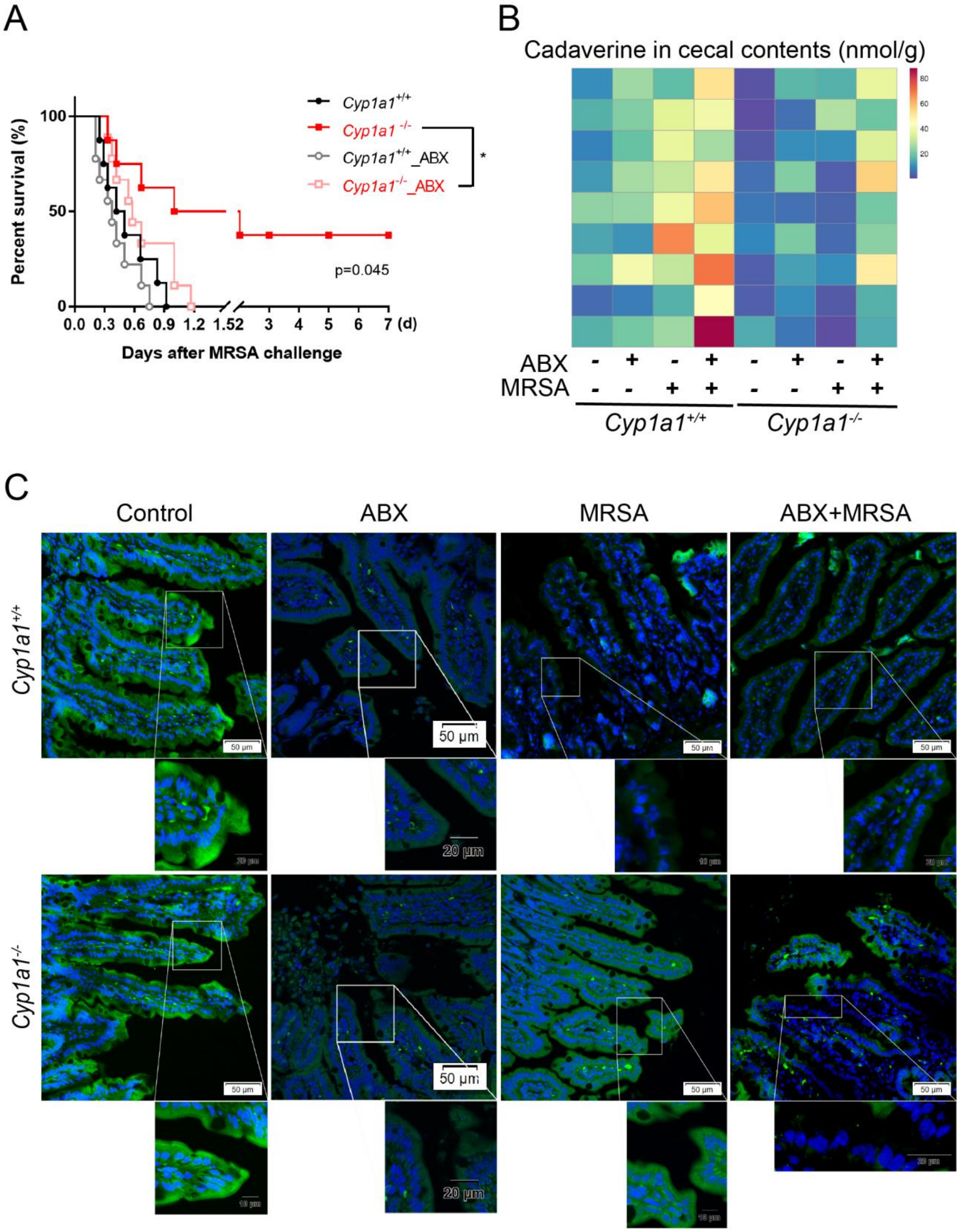

Supplementary Fig. 2 Spearman's correlation analysis.
Correlation analysis between the cadaverine level and paired *E. faecalis* abundance in the caecal contents from *Cyp1a1*$^{+/+}$ mice (**A**) and *Cyp1a1*$^{-/-}$ mice (**B**) with MRSA infection.

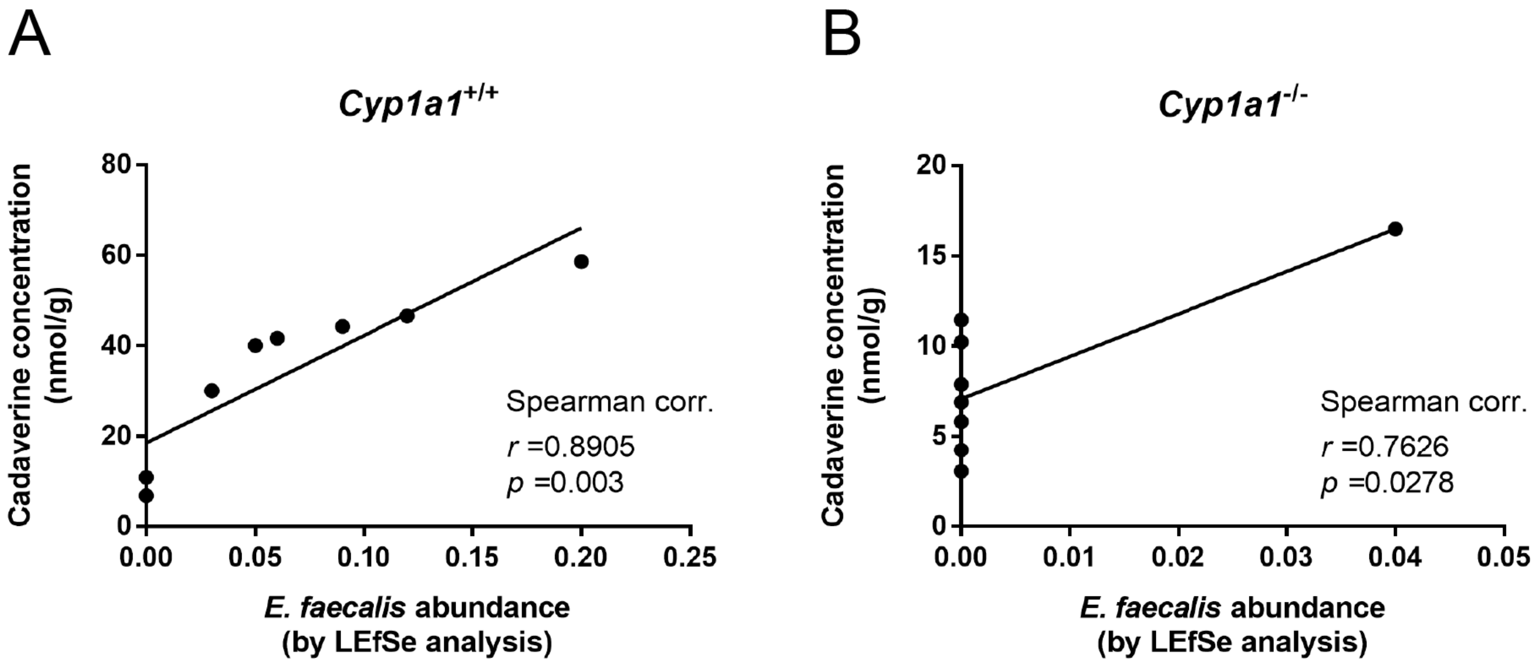

Supplement: Supplementary Figure 1 — Antibiotic administration reverses the protection of intestinal barrier integrity in Cyp1a1-knockout mice. (A) Survival of Cyp1a1+/+ (n = 8) and Cyp1a1–/– (n = 8) mice in the presence or absence of antibiotic cocktail (ABX) and subjected to MRSA from two independent experiments (*P < 0.05; log-rank analysis). (B) Heatmap of the caecal contents from each group. (C) Immunofluorescence staining of ileum sections for ZO-1 (green) and DAPI (blue) from each group (scale bar: 50 μm); white boxes indicate the magnified areas (scale bar: 20 μm). [file Data_Sheet_1.pdf]
